# A Single-Center Study on the Clinical Profiles and Ultrasonographic Assessments of Living Kidney Donors in the Marathwada Region of Maharashtra

**DOI:** 10.7759/cureus.53293

**Published:** 2024-01-31

**Authors:** Kshitija Gadekar, Rahul B Tengse, Saif Kibriya, Pranav R Kulkarni

**Affiliations:** 1 Nephrology, Mahatma Gandhi Mission (MGM) Medical College and Hospital, Aurangabad, IND

**Keywords:** bmi, ultrasonography, e-gfr, dtpa, donor kidney size

## Abstract

Living donor kidney transplantation plays a vital role in renal replacement therapy, particularly in India, where a substantial increase in kidney transplants has been observed. Thorough assessments of living kidney donors are crucial, focusing on parameters such as kidney size and glomerular filtration rate (GFR). Despite the importance of GFR in donor assessments, there is a noticeable lack of data on normal GFR ranges in the Indian population. This study aims to address the gap in knowledge by establishing a reference range for GFR in healthy kidney donors from the Marathwada region of Maharashtra. The research also explores the clinical profiles and ultrasonographic features of living kidney donors. A retrospective analysis was conducted at the Mahatma Gandhi Mission (MGM) Medical College and Hospital in Aurangabad, involving 134 living kidney donors. Inclusion criteria encompassed healthy donors with a BMI of less than 30 kg/m², while donors with uncontrolled hypertension, diabetes, microalbuminuria, or a measured GFR below 70 mL/min/1.73 m² were excluded. Comprehensive medical histories, demographic parameters, and ultrasonographic assessments were conducted, with GFR measured using 99M technetium diethylenetriamine pentaacetate scans. The study reveals that the majority of donors were females (80.6%), and the highest number fell within the 41-50 age group. Parents constituted the primary donor category (68.7%), reflecting a familial inclination toward organ donation. Ultrasonographic assessments indicated larger kidney sizes compared to other studies, suggesting regional or population-specific differences. The mean GFR for the right and left kidneys, as well as the total GFR, was within the expected range. The negative correlation between age and GFR emphasizes the need to consider age in donor assessments. The findings emphasize the unique features of this population, including a higher average age, female preponderance, and larger kidney sizes. The study contributes to the understanding of living kidney donors’ profiles in the region and highlights the importance of individualized assessments in the donor selection process.

## Introduction

Living donor kidney transplantation is an effective method of renal replacement treatment that enhances the quality of life of patients with end-stage renal disease [1,2]. In recent years, the number of kidney transplants in India has seen a significant increase from 2013 to 2018, with a total of 32,584 living donor kidney transplants and 5,748 deceased donor kidney transplants [3]. Thorough assessments are conducted to ensure the suitability of live kidney donors, with a focus on glomerular filtration rate (GFR) and kidney size estimation [4]. Methods such as creatinine clearance, estimated GFR (eGFR) formulae, or isotope scans are typically used to determine GFR, an important parameter in donor assessment protocols [5].

Effective living donation practices prioritize safety and positive outcomes for living kidney donors. This requires a comprehensive evaluation and risk assessment before donation, aligning with guidelines such as those provided by the Kidney Disease: Improving Global Outcomes (KDIGO) consortium [6]. These guidelines permit a donor’s GFR threshold to be as low as 60 ml/min/1.73 m². Notably, the British Transplantation Society 2018 [7] and the European Renal Best Practice 2013 [8] guidelines allow older donors to have a GFR below 60 ml/min/1.73m².

Despite the critical role of GFR in donor assessment, a noticeable gap exists in data on normal GFR ranges within the Indian population. Consequently, the primary objective of this research is to establish a reference range for GFR in healthy kidney donors from the Marathwada region of Maharashtra. This study endeavors to bridge this gap by investigating living kidney donors' clinical profiles and ultrasonographic features, focusing specifically on the Marathwada region of Maharashtra.

## Materials and methods

Study setting and participants

This retrospective study took place at a tertiary care center in the nephrology department of the Mahatma Gandhi Mission (MGM) Medical College and Hospital in Aurangabad, situated in the Marathwada region of Maharashtra. The study cohort comprised 134 living kidney donors who had undergone extensive medical and surgical evaluations. The study was approved by the MGM Medical College and Hospital Ethics Committee for Research on Human Subjects (approval number MGM/PHARMAC/ECRHS/2023/154).

Living kidney donor data was collected over the past 10 years from the records section of MGM Medical College and Hospital, and the data was analyzed from May 2023 to October 2023.

Participants in the study underwent thorough medical and surgical evaluations conducted by the nephrology department at MGM Medical College and Hospital. These evaluations were designed to be comprehensive, encompassing a detailed examination of the donor’s health to ensure their suitability for kidney donation. The focus was not only on routine health check-ups but also on a meticulous assessment of various factors influencing overall health.

The clinical profile, including age, gender, and medical history, was collected. GFR was measured using a 99M technetium diethylenetriamine pentaacetate (DTPA) scan, and the dimensions of the kidney were measured by ultrasonography.

Inclusion criteria

Healthy kidney donors who were near relatives of recipients (parents, siblings, spouses, and in-laws) with a BMI of less than 30 kg/m².

Exclusion criteria

Donors with uncontrolled hypertension, diabetes mellitus, or microalbuminuria with a measured GFR of less than 70 mL/min/1.73 m².

Data collection

Data collection involved obtaining a comprehensive medical history, demographic parameters (age and gender), and measurements of height, weight, and BMI. Ultrasonographic assessments were employed to measure kidney dimensions and gather relevant anatomical details.

Statistical analysis

The collected data was entered into Microsoft Excel 2019 (Microsoft Corporation, Redmond, WA, USA) and analyzed using IBM SPSS Statistics for Windows, Version 21.0 (Released 2012; IBM Corp., Armonk, NY, USA). All quantitative variables were described in mean and standard deviations, while qualitative data were described in frequency and percentages. Donors were categorized based on age and BMI, and the association with GFR, as measured by DTPA, was assessed using the Chi-square test. The Pearson correlation test was applied to find the correlation between age, BMI, and GFR; p < 0.05 is considered significant. Statistical analysis was performed using IBM SPSS Statistics for Windows, Version 21.0.

## Results

This study aims to contribute valuable insights into the clinical and ultrasonographic profiles of living kidney donors, with a specific focus on GFR in the Marathwada region of Maharashtra, India.

Sociodemographic details

The sociodemographic details of the study participants are shown in Table 1. The donors were stratified into six distinct age groups: <30, 31-40, 41-50, 51-60, 61-70, and >71 years. The age distribution revealed that the highest number of donors, comprising 47 individuals (35.1%), fell within the 41-50 age group. The mean age of the donors was 49.13 ± 10.75 years. There was a notable gender discrepancy, with a higher proportion of female donors (108, 80.6%) than male donors (26, 19.4%). The donors were categorized based on their relationship to the recipient, which included parents, siblings, spouses, in-laws, swap donors, and offspring, and it was found that the majority of donors were parents, accounting for 92 (68.7%) of the participants. There was a significant female preponderance, with a male-to-female ratio of 1:3.4.

**Table 1:** Sociodemographic details of the study participants

Sociodemographic details		Number	Percentage
Age group in years	<30	7	5.2
	31-40	20	14.9
	41-50	47	35.1
	51-60	39	29.1
	61-70	18	13.4
	>71	3	2.2
Gender	Female	108	80.6
	Male	26	19.4
Donor and recipient relationship	Parents	92	68.7
	Siblings	11	8.2
	Spouse	22	16.4
	In-laws	6	4.5
	Swap	2	1.5
	Offspring	1	0.7

BMI

The study divided donors into two groups based on their BMI: group A consisted of individuals with a normal weight (BMI of 18 to <24.99), while group B (BMI of >25) included those who were overweight or obese. The average BMI of the donors was 25.14 ± 1.67 kg/m². Out of the donors, the majority, accounting for 76 individuals (56.7%), had a BMI falling between the range of 25.0-29.9.

Kidney characteristics

Kidney Dimensions

Ultrasonographic assessments yielded measurements for kidney breadth and length, as shown in Table 2 and Table 3.

**Table 2:** Comparison of kidney length between right and left kidneys

Kidney length (in mm)	Right kidney, n (%)	Left kidney, n (%)
≤80	7 (5.2)	3 (2.23)
81-90	41 (30.3)	34 (25.37)
90-100	63 (47.2)	66 (49.25)
>100	23 (17.3)	31 (23.13)
Total	134 (100)	134 (100)

**Table 3:** Comparison of kidney breadth between the right and left kidneys

Kidney breadth (in mm)	Right kidney, n (%)	Left kidney, n (%)
≤30	7 (5.2)	3 (2.2)
31-35	29 (21.6)	17 (12.7)
36-40	55 (41.0)	37 (27.6)
41-45	28 (20.9)	42 (31.3)
46-50	9 (6.7)	25 (18.7)
>50	6 (4.4)	10 (7.5)
Total	134 (100)	134 (100)

Kidney Length

Table 2 illustrates the distribution of kidney lengths in millimeters, categorizing the data into four length ranges (≤80 mm, 81-90 mm, 91-100 mm, and >100 mm) for both the right and left kidneys. Overall, the distribution reveals a predominance of kidneys in the 91-100 mm length range, highlighting potential patterns or variations in kidney sizes within the studied population. The average length of the right kidney was 112.00 ± 7.34 mm, whereas the average length of the left kidney was 120.00 ± 7.36 mm.

Kidney Breadth

Table 3 outlines the distribution of kidney breadth in millimeters, categorizing the data into six distinct ranges for both the right and left kidneys. The majority of kidneys, constituting 55 (41.0%) on the right, have a breadth fall within the 36-40 mm range, while on the left side, 42 (31.3%) fall within the 41-45 mm range. The average width of the right kidney was 39.05 ± 6.09 mm, whereas the average width of the left kidney was 42.09 ± 7.81 mm.

GFR

GFR was assessed through a 99M technetium DTPA scan, providing valuable insights into renal function. Table 4 presents the distribution of GFR for both the right and left kidneys. GFR is categorized into four ranges (<40, 41-50, 51-60, and >60), and the data is provided in terms of counts and percentages for each category. The majority falls within the 41-50 GFR range, with 81 (60.4%) on the right and 85 (63.4%) on the left. The mean GFR for the right kidney was 49.45 ± 6.425 ml/min/1.73m², while for the left kidney it was 48.30 ± 6.405 ml/min/1.73m². The total GFR average is calculated at 97.75 ± 6.41 mL/min/1.73 m².

**Table 4:** Comparison of GFR between right and left kidneys GFR, glomerular filtration rate

GFR	Right kidney, n (%)	Left kidney, n (%)
<40	5 (3.7)	7 (5.2)
41-50	81 (60.4)	85 (63.4)
51-60	39 (29.1)	35 (26.1)
>60	9 (6.7)	7 (5.2)
Total	134 (100)	134 (100)

Correlation of age and GFR

**Figure 1:**
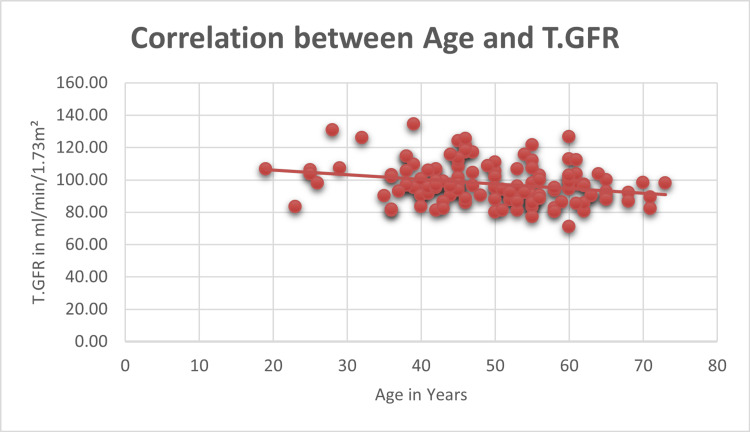
Correlation between age and T.GFR p < 0.05 is considered significant T.GFR, total glomerular filtration rate

As shown in Figure 1, the study investigated the relationship between age and GFR as measured by DTPA scan, and the correlation analysis revealed a statistically significant moderate negative correlation (r = -0.253, p = 0.003) between age and total GFR (T.GFR) among the 134 participants. This negative correlation indicates a moderate inverse relationship, suggesting that as individuals age, their T.GFR tends to decrease.

Table 5 presents a detailed distribution of GFR in ml/min/1.73 m² among different age groups, providing a comprehensive overview of renal function within each category. The highest GFR was observed in the age group <30 years, while the lowest was in those aged over 70 years. A Chi-square test applied as a measure of association for the age category of donors versus eGFR from the DTPA scan yielded a statistically significant result (p < 0.05). This indicates that there is a significant association between age categories and eGFR, reinforcing the link between age and renal function as assessed by the DTPA scan.

**Table 5:** Comparison of GFR across different age groups GFR, glomerular filtration rate

Age in years	GFR in ml/min/1.73 m²			
	60-79.9, n (%)	80-99.9, n (%)	>100, n (%)	Total, n (%)
<30	0	2 (29.6)	5 (71.4)	7 (100)
31-50	1 (1.49)	38 (56.71)	28 (41.7)	67 (100)
51-70	2 (3.50)	45 (73.68)	13 (22.80)	60 (100)
Total	3 (2.2)	85 (63.4)	46 (34.3)	134 (100)

Correlation of BMI and GFR

**Figure 2:**
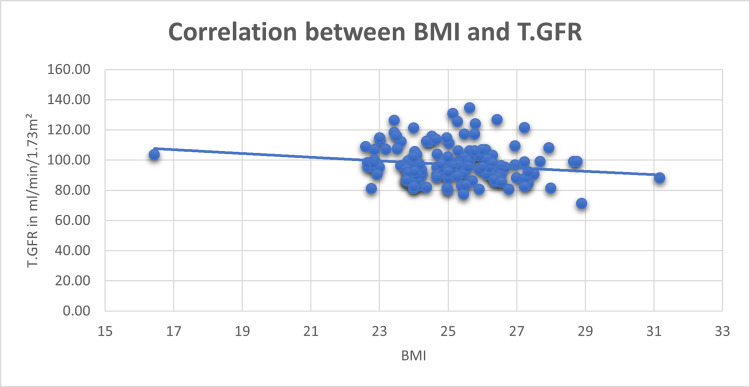
Correlation between BMI and T.GFR p < 0.05 is considered significant T.GFR, total glomerular filtration rate

As illustrated in Figure 2, the analysis indicates a statistically nonsignificant correlation (r = -0.156, p = 0.072) between BMI and T.GFR among the 134 participants. The weak and nonsignificant negative correlation implies that, within this sample, the relationship between BMI and T.GFR lacks strength and may be influenced by other factors. The Chi-square test further supports this finding, revealing no significant association between BMI and GFR (p > 0.05). This suggests that, in this particular study, BMI does not appear to be a significant determinant of GFR.

## Discussion

This study, conducted in the Marathwada region of Maharashtra, provides a comprehensive evaluation of living kidney donors, focusing on diverse demographic, clinical, and ultrasonographic parameters. The study involves 134 living donors, predominantly females, and offers insights into the correlations between age, gender, BMI, kidney dimensions, and GFR. According to previous studies [5,9,10], factors like GFR, proteinuria, hematuria, cysts, stones, genetics, and a family history of kidney disease are crucial considerations involved in evaluating living kidney donors.

The age distribution of the donors reveals a significant predominance in the 41- to 50-year-old age group, with a mean age of 49.13 ± 10.75 years among donors. This finding contrasts with results from other studies in India [11-13], where the mean age was lower. The observed higher age in our study prompts a need for further exploration into age-related considerations in the context of living kidney donation.

Additionally, the study observes a higher proportion of female donors, with a male-to-female ratio of 1:3.4. This gender distribution trend aligns with previous studies in Kerala [12] and Maharashtra [13], but is contrary to findings from Kenya [14], where the majority of the donors are males (61%). Our findings emphasize the need for increased awareness among male donors about organ donation.

The majority of donors being parents or spouses suggests a familial inclination toward organ donation. This aligns with other studies emphasizing the prevalence of first-degree relatives as donors [13-15]. Our study suggests that encouraging awareness among male spouses could further enhance organ donation rates, reflecting the need for targeted interventions.

It was observed that the mean length of the right donor kidney was 112 ± 7.34 mm, whereas, for the left donor kidney, it measured 120 ± 7.36 mm. These findings contrast with those of a study by Srivastava et al. in North India [11], where the right kidney measured 95.3 ± 8.47 mm and the left kidney measured 99 ± 9.71 mm. Also, the disparities in the findings are noted in comparison to the study by Muthusami et al. [16], where it was 96 + 9.7 mm for the right kidney and 97.1 ± 8.9 mm for the left kidney. The observed bulkier left kidney in our study suggests the possibility of racial differences influencing anatomical characteristics.

Our study findings reveal that the mean breadth for the right kidney was 39.05 ± 6.09 mm, and for the left kidney, it was 42.09 ± 7.81 mm. The measurements were found to be less than the findings from Muthusami et al. [16], where the right kidney had a breadth of 45 ± 7 mm and the left kidney had a breadth of 45.4 ± 6.3 mm. These differences in kidney length and breadth emphasize potential racial or regional variations in anatomical characteristics. The observed variations highlight the necessity of considering population-specific characteristics in the evaluation of living kidney donors.

The GFR measurements obtained through the 99M technetium DTPA scan serve as a robust indicator of renal function in our study. The mean GFR values for both the right and left kidneys, as well as the total GFR, fall within the expected range for the study participants. The measured GFR, a crucial parameter in donor evaluation, is within the midrange of 97.75 ± 6.41 mL/min/1.73 m². The detailed breakdown by age groups further highlights variations in GFR across different donor age categories.

The highest eGFR was observed in the age group <30, while the lowest was among individuals aged more than 70 years. The average eGFR in our study aligns with findings from other studies, indicating adequacy against established thresholds. In a study conducted by Sheetal et al. in Kerala, the reported average eGFR was 84.51 ± 1.50 ml/min/1.73m² [12]. Similarly, Chavan et al., in a study in Pune, reported average eGFR values of 103.83 ± 10.07 ml/min/1.73m² [13]. Additionally, the study by Bahirani et al. reported an average eGFR of 99.47 ± 14.4 ml/min/1.73m² [17], and in the study by Lin et al., it was 112.8 mL/min/1.73 m² [18].

The study reports a mean BMI of 25.14 ± 1.67 kg/m², which aligns with findings from similar studies conducted in India [12,13]. However, the study did not find a significant association between BMI and GFR, suggesting that kidney function might not be significantly affected by variations in BMI within the observed range. These results emphasize the complexity of the relationship between BMI and renal function, suggesting that other factors may play a more substantial role in determining GFR.

Comparisons with other studies revealed notable variations in demographic and clinical parameters. The Marathwada region demonstrated unique characteristics, such as higher average age, female preponderance, and larger kidney sizes, suggesting the influence of regional and population-specific factors. Understanding these regional differences is crucial for informed medical decision-making and the development of targeted health interventions within the local population.

Limitation

While the study provides valuable insights into living kidney donors in the Marathwada region, its limited sample size may constrain the generalizability of the findings. The absence of a control group and the utilization of a single-center approach might limit external validity. To enhance the robustness and applicability of the conclusions, future research endeavors should consider employing larger and more diverse samples, incorporating control groups, and adopting multicenter approaches. These measures can help to ensure that the findings are more generalizable and representative of the population.

## Conclusions

The study provides valuable insights into the demographic and clinical characteristics of living kidney donors in the Marathwada region of Maharashtra. The Marathwada region demonstrated unique characteristics, such as higher average age, female preponderance, and larger kidney sizes, suggesting the influence of regional and population-specific factors. The correlation between age and GFR suggests a direct relationship, emphasizing the importance of considering age in donor assessments. However, no significant association was found between BMI and GFR. The study contributes to understanding living kidney donors’ profiles in the region, emphasizing the need for individualized assessments in the donor selection process.
